# Novel GLP-1 Analog Supaglutide Reduces HFD-Induced Obesity Associated with Increased Ucp-1 in White Adipose Tissue in Mice

**DOI:** 10.3389/fphys.2017.00294

**Published:** 2017-05-15

**Authors:** Yun Wan, Xi Bao, Jiabao Huang, Xiangyu Zhang, Wenjuan Liu, Qiaoli Cui, Dongdong Jiang, Zhihong Wang, Rui Liu, Qinghua Wang

**Affiliations:** ^1^Department of Endocrinology and Metabolism, Huashan Hospital, Fudan UniversityShanghai, China; ^2^Yinnuo Pharmaceutical Technology Co. Ltd.Shanghai, China; ^3^Division of Endocrinology and Metabolism, Keenan Research Centre for Biomedical Science, St. Michael's HospitalToronto, ON, Canada; ^4^Departments of Physiology and Medicine, Faculty of Medicine, University of TorontoON, Canada

**Keywords:** GLP-1 analog, supalgutide, obesity, diabetes, Ucp1

## Abstract

GLP-1, an important incretin hormone plays an important role in the regulation of glucose homeostasis. However, the therapeutic use of native GLP-1 is limited due to its short half-life. We recently developed a novel GLP-1 mimetics (supaglutide) by genetically engineering recombinant fusion protein production techniques. We demonstrated that this formulation possessed long-lasting GLP-1 actions and was effective in glycemic control in both type 1 and type 2 diabetes rodent models. Here, we investigated the effects of supaglutide in regulating energy homeostasis in obese mice. Mice were fed with high-fat diet (HFD) for 6 months to induce obesity and then subjected to supaglutide treatment (300 μg/kg, bi-weekly for 4 weeks), and placebo as control. Metabolic conditions were monitored and energy expenditure was assessed by indirect calorimetry (CLAMS). Cold tolerance test was performed to evaluate brown-adipose tissue (BAT) activities in response to cold challenge. Glucose tolerance and insulin resistance were evaluated by intraperitoneal glucose tolerance test and insulin tolerance tests. Liver and adipose tissues were collected for histology analysis. Expression of uncoupling protein 1(Ucp1) in adipose tissues was evaluated by Western blotting. We found that supaglutide treatment reduced body weight, which was associated with reduced food intake. Compared to the placebo control, supaglutide treatment improved lipid profile, i.e., significantly decreased circulating total cholesterol levels, declined serum triglyceride, and free fatty acid levels. Importantly, the intervention significantly reduced fatty liver, decreased liver triglyceride content, and concomitantly ameliorated liver injury exemplified by declined hepatic alanine aminotransferase (ALT) and aspartic transaminase (AST) content. Remarkably, supaglutide reduced hepatic lipid accumulation and altered morphometry in favor of small adipocytes in fat. This is consistent with the observation that supaglutide increased tolerance of the mice to cold environment associated with up-regulation of Ucp1 in the inguinal fat. Furthermore, supaglutide improved glucose tolerance, and insulin sensitivity in the obese mice suggesting improved glucose and energy homeostasis. Our findings suggest that supaglutide exerts beneficial effect on established obesity through reducing energy intake and is associated with brown remodeling of white adipose tissue.

## Introduction

Glucagon-like peptide-1 (GLP-1) is an incretin hormone secreted by gastrointestinal L cells in response to nutrient ingestion Meier and Nauck (2005). It exerts numerous biological functions including stimulating glucose-dependent insulin secretion, inhibiting glucagon release, inducing satiety and slowing gastric emptying (Rajeev and Wilding, 2016). The physiological properties of GLP-1 render it as an ideal therapy for obesity and type 2 diabetes (T2D) (Rajeev and Wilding, 2016). However, native GLP-1 is not suitable for therapeutic use due to its short-circulating half-life (*t*1/2 < 2 min), which results mainly from rapid enzymatic inactivation by dipeptidyl peptidase-IV (DPP-IV), and/or rapid kidney clearance (Kieffer et al., 1995). In order to prolong the action of GLP-1, great efforts have been made in the past decade and two strategies are accessible: one is the use of DPP-IV inhibitors and the other is the development of DPPIV-resistant analog through structural modifications which prevent DPP-IV degradation (Drucker et al., 2010, 2011; Lindamood and Taylor, 2015; Lee, 2016; Rajeev and Wilding, 2016).

We developed a GLP-1 analog supaglutide by fusing a pair of GLP-1 molecules with immunoglobulin constant region that contains partial hinge chains (Kumar et al., 2007; Wang et al., 2010). While the fusion chimera displayed high avidity to the GLP-1 receptor, pharmacokinetic studies showed that a single intraperitoneal injection of supaglutide led to rapid increase in drug concentration in circulation and maintaining at high levels for more than 1 week (Wang et al., 2010). The long lasting effects of supaglutide were probably due to its large molecular mass (~60 kD) which made it not easily cleared by kidney (Wang et al., 2010). Moreover, the fusion protein appeared relatively resistant to DPPIV degradation (Wang et al., 2010) and possibly other degrading enzymes (Hupe-Sodmann et al., 1995), as exemplified by both *in vitro* and *in vivo* studies (Wang et al., 2010). Notably, supaglutide has biological relevance since it showed high binding affinity to GLP-1 receptor and stimulated insulin secretion in a glucose-dose dependent manner in insulin-secreting INS-1 cells (Wang et al., 2010). Supaglutide has long-lasting effects on the modulation of glucose homeostasis, exemplified by the observations that the mice displayed significantly reduced glucose excursion as determined by intraperitoneal glucose tolerance test (IPGTT) performed 1 week after a single injection (Wang et al., 2010). Importantly, supaglutide and its relevant constructs are found to be effective in reducing the incidence of diabetes in multiple-low-dose streptozotocin (STZ)-induced type 1 diabetes and db/db type 2 diabetes in mice (Kumar et al., 2007; Soltani et al., 2007; Wang et al., 2010). One mechanism underlying the protective role of GLP-1 in diabetes is its insulinotrophic and beta cell protective actions which comply with GLP-1 therapeutic effects in the treatment of diabetes. However, limited data is available in term of this GLP-1 analog on energy homeostasis.

Here, we investigated whether supaglutide exerts regulatory effects on energy metabolism using murine obese model induced by high fat diet (HFD) feeding. We showed that twice a week injection of supaglutide exerted body weight-sparing effects and improved glucose tolerance and insulin sensitivity in HFD-induced obese mice. Moreover, supaglutide-treated mice were more tolerant to cold exposure and demonstrated upregulated thermogenic uncoupling protein 1(Ucp1) expression in the inguinal white fat. Our results imply that supaglutide may serve as an alternative potent GLP-1 therapy for obesity, type 2 diabetes, and other metabolic diseases.

## Methods

### Animals

Adult male C57BL/6 mice (Slack Laboratory Animal, Shanghai, China), CD-1 mice (Vital River Laboratory Animal Technology Co., Ltd., Beijing, China), and db/db mice (CAVENS Lab Animal Technology Co., Ltd., Changzhou, China) were housed under controlled temperature conditions and a 12 h light/12 h dark cycle with free access to food and water except where noted. To produce diet-induce obesity, 5-month-old mice were fed with HFD (calorie density: 60% total kilocalories from fat and 20% from carbohydrate; Product number: D12492; research Diets, New Brunswick, USA) and plain water for 6 months. Before treatment, body weight, fasting blood glucose, IPGTT, intraperitoneal insulin tolerance testing (IPITT), and food intake were measured as baseline data. Treatment study was performed by intraperitoneally injecting supaglutide (Yinnuo Pharmaceutical Technology Co. Ltd., 300 μg/kg) twice a week (PBS as control) for 4 weeks. Animal care procedures were approved by the Animal Care and Use Committee of the Fudan University Shanghai Medical College and followed the National Institute of Health guidelines on the care and use of animals.

### Intra peritoneal glucose tolerance test, insulin tolerance test

For IPGTT, mice were fasted overnight for 16 h, and were given 2 g glucose/kg body weight via intra-peritoneal (i.p.) injection. Blood was drawn from the tail vein and glucose levels were measured using a glucometer (Bayer, German) at 0, 15, 30, 60, 90, and 120 min after glucose administration.

For ITT, mice were fasted for 6 h, and were i.p. injected with recombinant human insulin (Novo Nordisk, Danish) (1.5 U/kg), blood glucose levels were measured at 0, 10, 20, 30, 60, and 120 min after insulin administration.

### Tissue histology

Tissues fixed in 4% paraformaldehyde were sectioned after being paraffin embedded. Multiple sections were prepared and stained with hematoxylin and eosin (HE) for general morphological observations.

### Serology studies

Blood samples were centrifuged at 2,000 g for 10 min to obtain serum. Triglyceride (TG), Total cholesterol (TC), high density lipoprotein (HDL), low-density lipoprotein (LDL), aspartate aminotransferase (AST), and alanine aminotransferase (ALT) levels were analyzed using Zhuoyue-450 automatic biochemical analysis device (Shanghai Kehua Biotechnology, China). Nonesterified fatty acids (NEFA) levels were determined using the LabAssay™ NEFA kit (WAKO, Japan).

### Indirect calorimetry

Metabolic rates were measured by indirect calorimetry in mice 4 weeks after treatment by using the Comprehensive Lab Animal Monitoring System (CLAMS, Columbus Instruments, USA). Mice were maintained at 24°C under a 12-h light/dark cycle. Food and water is available *ad libitum*. Mice were acclimatized to individual cages for 24 h before recording, and then underwent 24 h of monitoring. Each cage was monitored for metabolic parameters (including oxygen consumption and carbon dioxide production) at 25-min intervals throughout the 48 h period. Parameters of oxygen consumption (mL/h) and carbon dioxide production (mL/h) were recorded for each mouse. Heat production (energy expenditure) was calculated as described previously (Tseng et al., 2008).

### Western blotting

Immunoblotting was performed as previously described (Wan et al., 2015). Primary antibody Ucp1 (1:500 for white adipose tissues and 1:4,000 for brown adipose tissue) was from Abcam (ab155117), HSP90 (1:1,000) was from Santa Cruz (sc-7947), and GAPDH (1:1,000) was from Biotech Well (WB0197). Horseradish peroxidase-conjugated secondary antibody (1:5,000) was from SAB (anti-Rabbit 3012; anti-Mouse: 3032). Protein band densities were quantified using the Image J program.

### Cold exposure test

Mice after 4 weeks' treatment were starved for 6 h, followed by being placed for 6 h in a room with a temperature of 4–8°C. Body temperature was recorded once per hour with a rectal probe connected to a digital thermometer.

### Statistical analysis

All data were presented as means ± SEM. Data analyzes were carried out with the Graph-Pad Prism 5 program. Paired Student's *t*-test, unpaired Student's *t*-test, or two-way repeated measures (RM) ANOVA was used for direct comparisons between groups where applicable. Differences were considered statistically significant at *p* < 0.05 level.

## Results

### Induction of obesity in mice

**Figure 1:**
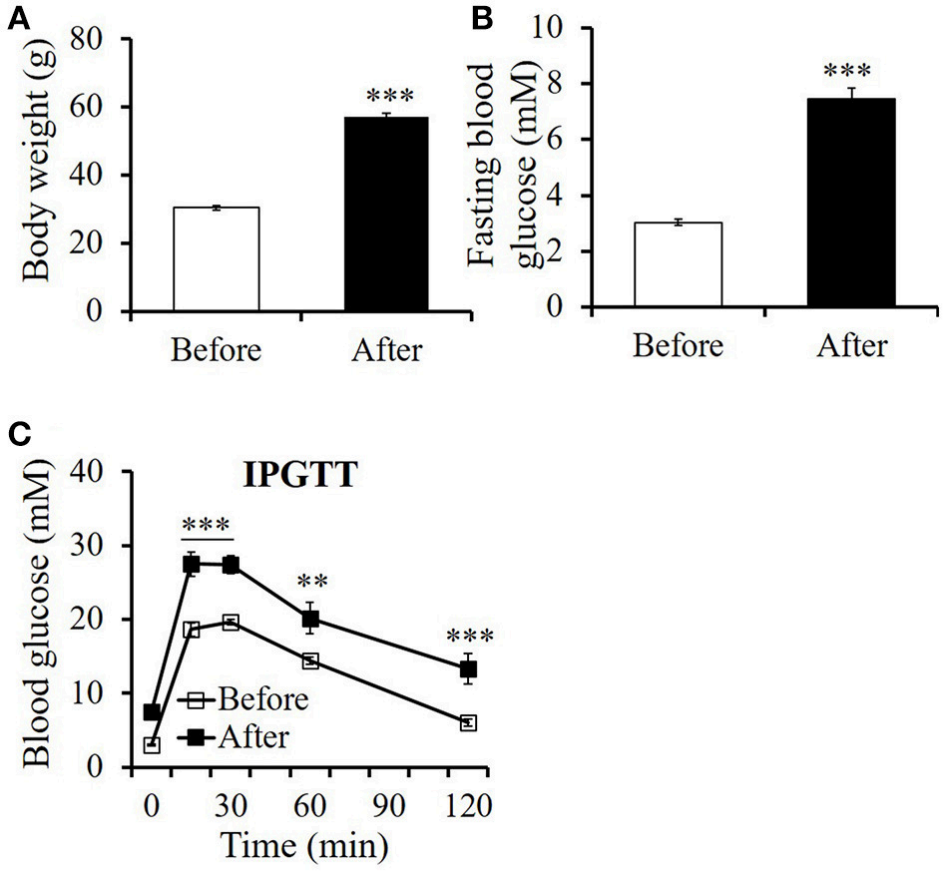
**High-fat diet feeding induced remarkable obesity in mice. (A)** Body weight, **(B)** fasting blood glucose level, and **(C)** glucose tolerance assayed by an intraperitoneal glucose tolerance test (IPGTT) before and after 6 months' high-fat diet feeding. All values are means ± SE (*n* = 11 for each group). Differences of **(A)** body weight, **(B)** fasting blood glucose between before and after HFD feeding were analyzed using paired Student's *t*-test method. Differences of **(C)** blood glucose levels at indicated time point during IPGTT between before and after HFD feeding is analyzed using two-way RM ANOVA method. ^**^p < 0.01; ^***^p < 0.001.

To produce diet-induced obesity, male C57BL/6 mice were fed with HFD (60% fat and 20% carbohydrate). After 6-month feeding of HFD, the mice developed obvious obesity (Figure 1A) associated with elevated fasting blood glucose levels (Figure 1B) and impaired glucose tolerance (Figure 1C).

### Supaglutide attenuates body weight gain in HFD-induced obese mice

**Figure 2:**
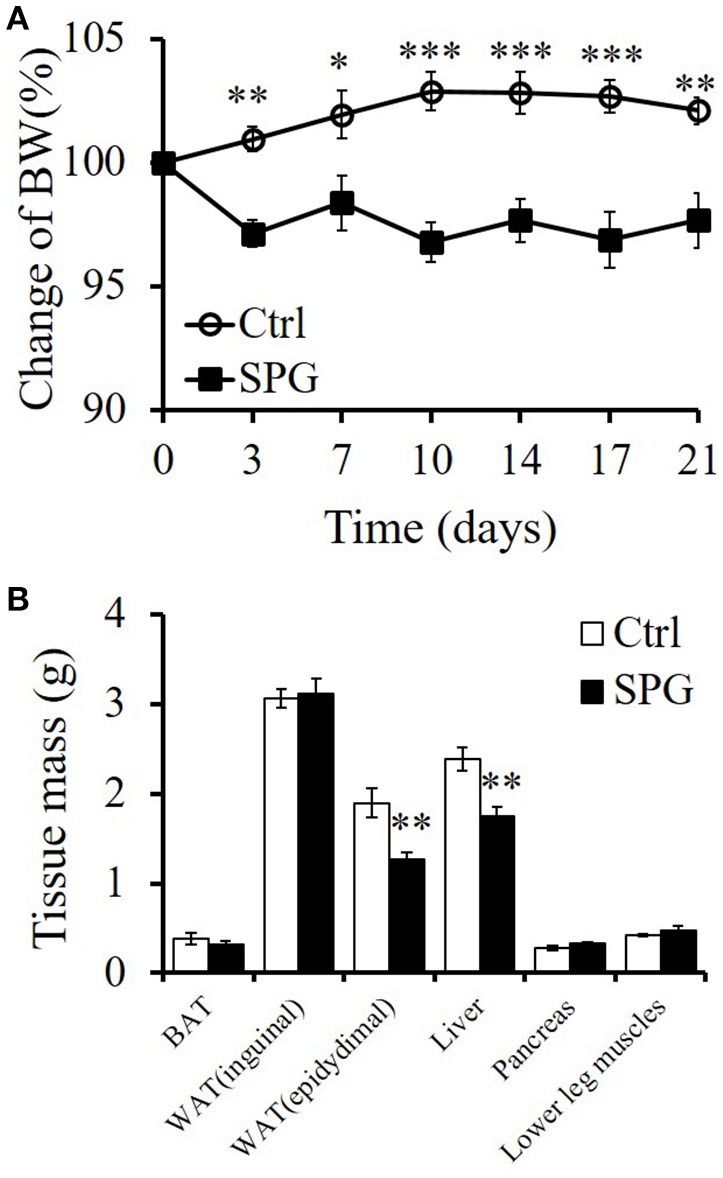
**Supaglutide attenuates body weight gain in DIO mice. (A)** Body weight gain of the PBS- and supaglutide- treated mice was recorded and expressed as the percentage of weight at the day before first injection. Significant difference between control and SPG is determined using two-way RM ANOVA method. **(B)** Weight of tissues. Significant difference between control and SPG is determined using unpaired Student's *t*-test. All values are means ± SE (*n* = 5 for each group). ^*^p < 0.05; ^**^p < 0.01; ^***^p < 0.001.

To examine whether supaglutide attenuates obesity, HFD-induced obese mice (body weight >50 g) were randomized grouped and injected without (control) or with supaglutide (300 μg/kg) twice a week; the body weights of the mice were measured at the day of injection. Under HFD feeding conditions, while untreated obese mice continue to yield their body weight, supaglutide treatment significantly decreased their body weight in obese mice (Figure 2A). The weight-sparing effects were observed 3 days after the first injection, and the supaglutide-treated mice kept leaner throughout the experiment.

The weight reducing effects of supaglutide was found to be associated with the reduction in part of visceral fat, specifically, the epididymal fat (epididymal fat: SPG vs. Ctrl = 2.45 ± 0.06% vs. 3.67 ± 0.17%, *p* < 001) and the mass of liver (SPG vs. Ctrl = 3.43 ± 0.04% vs. 4.57 ± 0.05%, *p* < 001) (Figure 2B). Interestingly, the mass in relation to body weight of other tissues examined, including brown fat, inguinal fat (which represents part of subcutaneous fat), pancreas and lower leg muscles, were not significantly changed (Figure 2B, *p* > 0.05).

### Supaglutide improves hepatic steatosis and reduces adipocyte size in the fat depots

**Figure 3:**
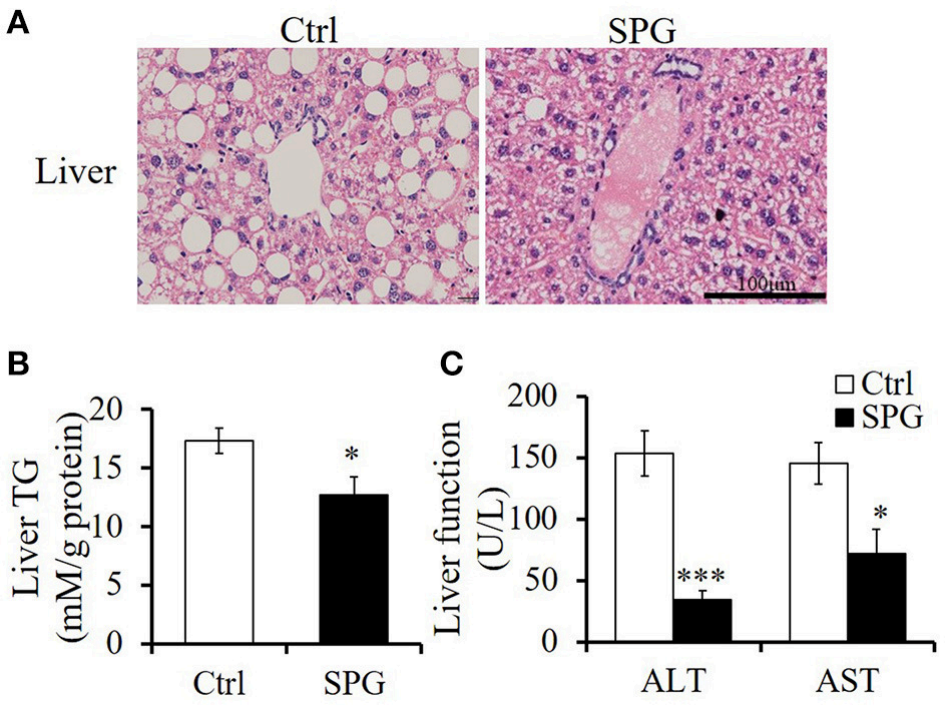
**Supaglutide improves hepatic steatosis of the DIO mice. (A)** H&E staining of liver tissues. Scale bar: 100 μm, 400×. **(B)** Triglyceride content in liver tissue was determined and normalized to the total protein in the samples. **(C)** Aspartate aminotransferase (AST) and alanine aminotransferase (ALT) levels were measured in the blood serum of the mice. Significant difference between control and SPG is determined using unpaired Student's *t*-test. All values are means ± SE (*n* = 5 for each group). ^*^p < 0.05; ^***^p < 0.001.

**Figure 4:**
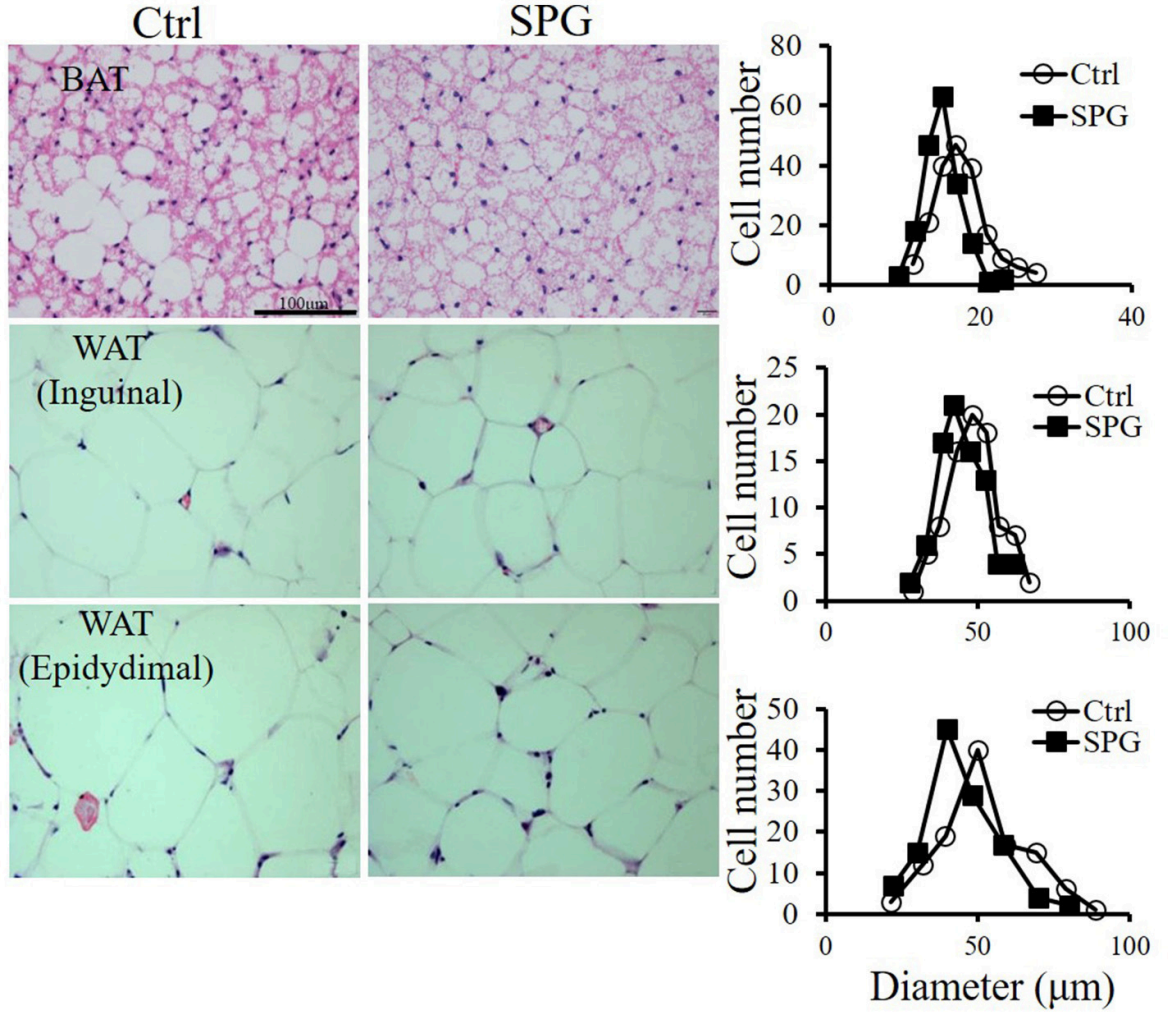
**Supaglutide reduces lipid accumulation in the adipose tissues. Left**, H&E staining of BAT, inguinal WAT and epididymal WAT; **right** panel, distributions of adipocyte cell size (Data were collected from H&E-stained sections from five individual mice, five fields per mouse, 5–10 cells per field in each group, using Image J software). Scale bar: 100 μm, 400×.

Supaglutide injections twice a week for 4 weeks drastically reduced ectopic lipid accumulation in liver of the obese mice on HFD feeding (Figure 3A). This was associated with significantly decreased liver triglyceride contents compared with the non-treated obese control mice (Figure 3B, SPG vs. Ctrl = 12.7 ± 0.7 vs. 17.32 ± 0.47, *p* < 0.05). Furthermore, while the untreated control mice exhibited impaired liver function caused by HFD feeding as reflected by high ALT and AST content, supaglutide treatment significantly decreased the levels of the two enzymes (Figure 3C, ALT: SPG vs. Ctrl = 34.2 ± 7.7 vs. 153.4 ± 18.7, *p* < 0.01; AST: SPG vs. Ctrl = 72.20 ± 19.29 vs. 145.6 ± 16.8, *p* < 0.05). Figure 4 shows the morphologies of the brown adipose tissue (BAT), inguinal white adipose tissue (WAT) and epididymal WAT. Cell sizes in all fat depots tended to be smaller in the supaglutide group compared with the controls.

### Supaglutide improves lipid profile in the obese mice

**Figure 5:**
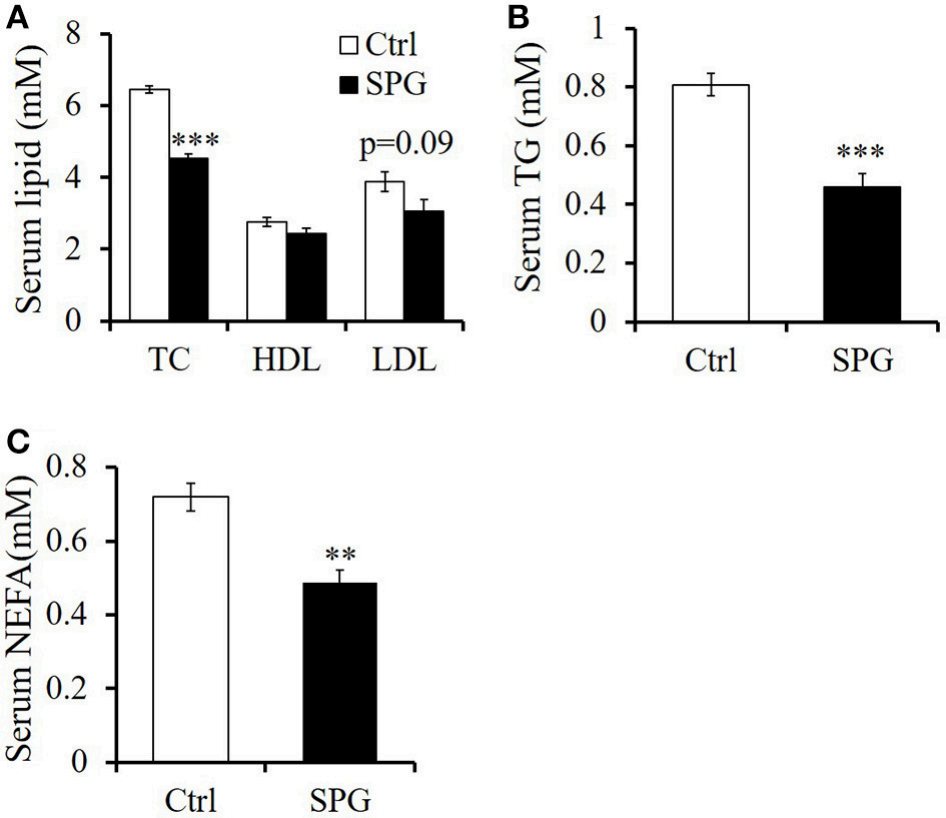
**Supaglutide improves lipid profile in the DIO mice. (A)** Total cholesterol (TC), high density lipoprotein (HDL), and low-density lipoprotein (LDL). **(B)** Triglyceride (TG). **(C)** Nonesterified fatty acids (NEFA). Significant difference between control and SPG is determined using unpaired Student's *t*-test. All values are means ± SE (*n* = 5 for each group). ^**^p < 0.01; ^***^p < 0.001.

Serology studies revealed that supaglutide treatment significantly decreased circulating levels of TC by 30% (Figure 5A, *p* < 0.001), and although not statistically significant, supaglutide also decreased circulating LDL levels by 21% (Figure 5A, *p* = 0.09). Moreover, supaglutide treatment decreased the TG level by 57% (Figure 5B, *p* < 0.001) and NEFA level by 68% (Figure 5C, *p* < 0.01) compared with control.

### Supaglutide does not increase energy expenditure significantly but upregulates Ucp1 protein expression in the inguinal fat

**Figure 6:**
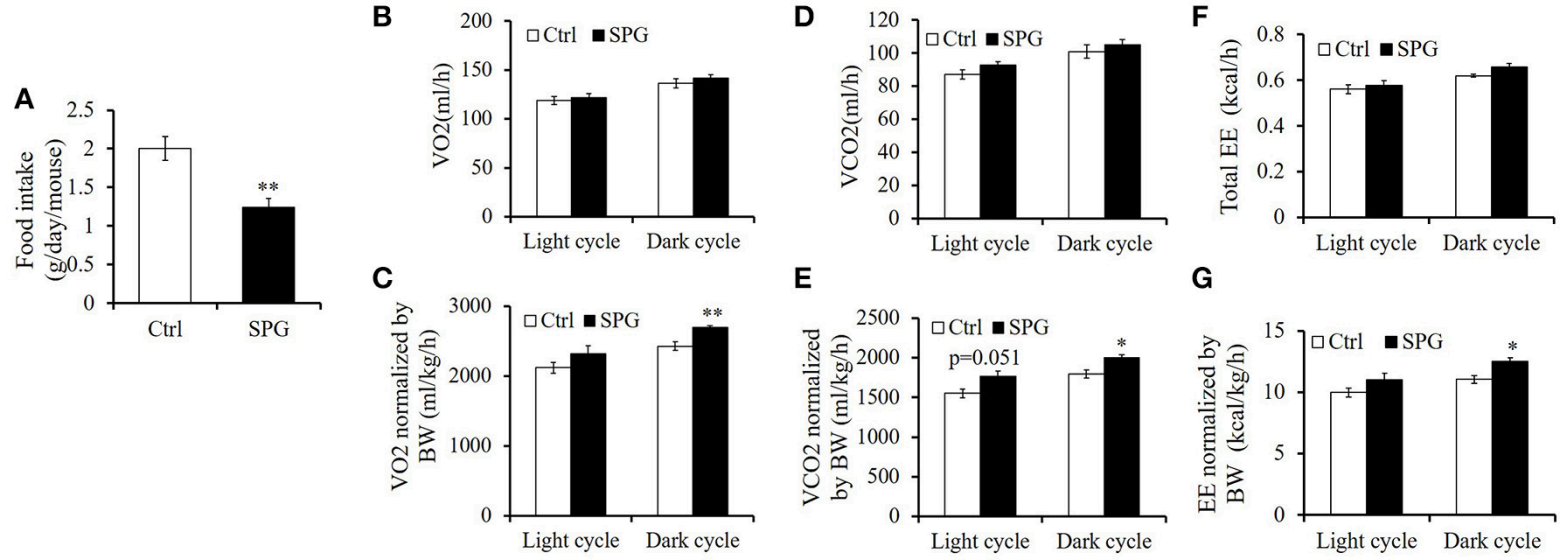
**Effect of supaglutide on food intake and energy expenditure in the DIO mice. (A)** Food intake. Oxygen consumption (VO2) expressed as per mouse in **(B)** and normalized by body weight (BW) in **(C)**. Carbon dioxide release (VCO2) expressed as per mouse in **(D)** and normalized by BW in **(E)**. Energy expenditure (EE) expressed as per mouse in **(F)** and normalized by BW in **(G)**. Significant difference between control and SPG is determined using unpaired Student's *t*-test. All values are means ± SE (*n* = 4 for each group). ^*^p < 0.05; ^**^p < 0.01.

In accord with GLP-1's capability in regulating gastric empting and food intake, supaglutiude treated mice showed reduction in body weight which was associated with reduced food intake significantly (Figure 6A, SPG vs. Ctrl = 1.24 ± 0.00 vs. 2.00 ± 0.05 g/day, *p* < 0.01). To determine whether supaglutide affected energy expenditure, we measured metabolic rate by indirect calorimetry using Comprehensive Lab Animal Monitoring System (CLAMS). As shown in our study, VO2 (Figure 6B), VCO2 (Figure 6D), and energy expenditure (EE) (Figure 6F) expressed as per-whole-mouse showed a trend, but not statistically different, of increase in supaglutide group. When normalized by body weight, VO2, VCO2, and EE of the supaglutide-treated mice demonstrated significant increase in the dark cycle (Figures 6C,E,G).

**Figure 7:**
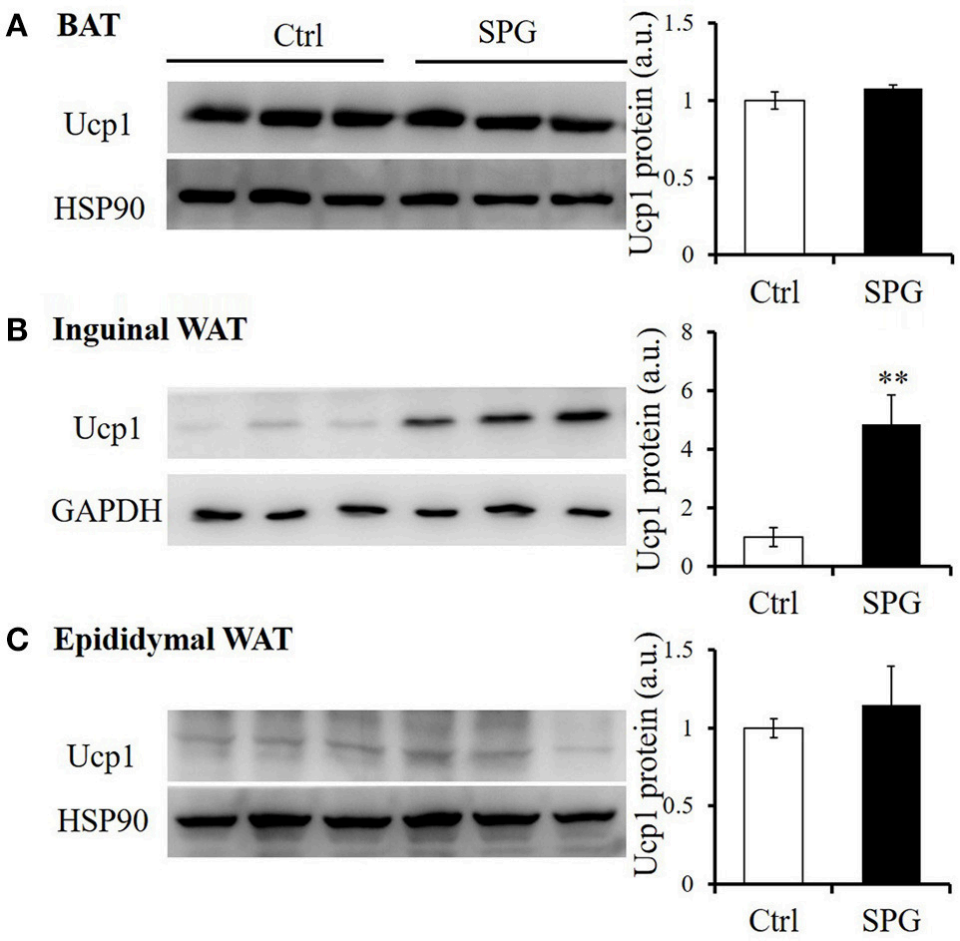
**Supaglutide upregulates Ucp1 protein expression in inguinal WAT**. Ucp1 protein expression in **(A)** BAT, **(B)** inguinal WAT, and **(C)** epididymal WAT was determined by WB. The bar graph represents quantitative results of 2–3 assays from 5 mice. Significant difference between control and SPG is determined using unpaired Student's *t*-test. ^**^p < 0.01.

It has been reported that central injection of GLP-1 promoted BAT thermogenesis, leading to increased energy expenditure (Lockie et al., 2012; Beiroa et al., 2014). We thus examined if peripheral injection of supaglutide enhanced BAT activity in the obese mice. Ucp1 is generally considered as a marker of BAT activity because it is highly expressed in BAT and dissipates the proton gradient across the mitochondrial inner membrane to produce heat (Stanford et al., 2013). Interestingly, we found that although supaglutide had no effect on Ucp1 expression in BAT (Figure 7A) and epidydimal WAT (Figure 7C), it significantly upregulated the expression of Ucp1 in the inguinal WAT (Figure 7B). This suggests that supaglutide does not enhance BAT thermogenesis but promote browning of inguinal white adipose tissue.

### Supaglutide increases tolerance of obese mice with cold exposure

**Figure 8:**
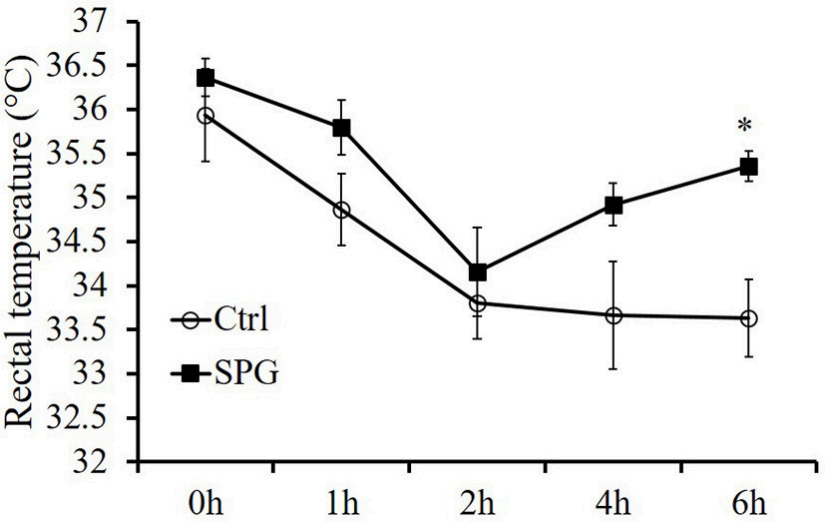
**Supaglutide-treated mice were more tolerant with cold exposure**. Mice after 4 weeks' treatment were starved for 6 h, followed by being placed for 6 h in a room with a temperature of 4–8°C. Body temperature was recorded once per hour with a rectal probe connected to a digital thermometer and the significant difference is indicated for control vs. SPG by two-way RM ANOVA. All values are means ± SE (*n* = 5 for each group). ^*^p < 0.05.

Upregulation of Ucp1 protein is a key element in maintaining core body temperature, especially when the body exposed to cold environment (Cannon and Nedergaard, 2004). Here, we found the drug treated-mice had a trend of higher core body temperature when housed in room temperature and kept higher rectal temperature when they were exposed to cold environment, displaying activated thermogenesis compared with the control group (Figure 8). This result shows that supaglutide was able to increase body adaptation to cold exposure by generating more heat.

### Supaglutide improves glucose tolerance and insulin sensitivity in the obese mice

**Figure 9:**
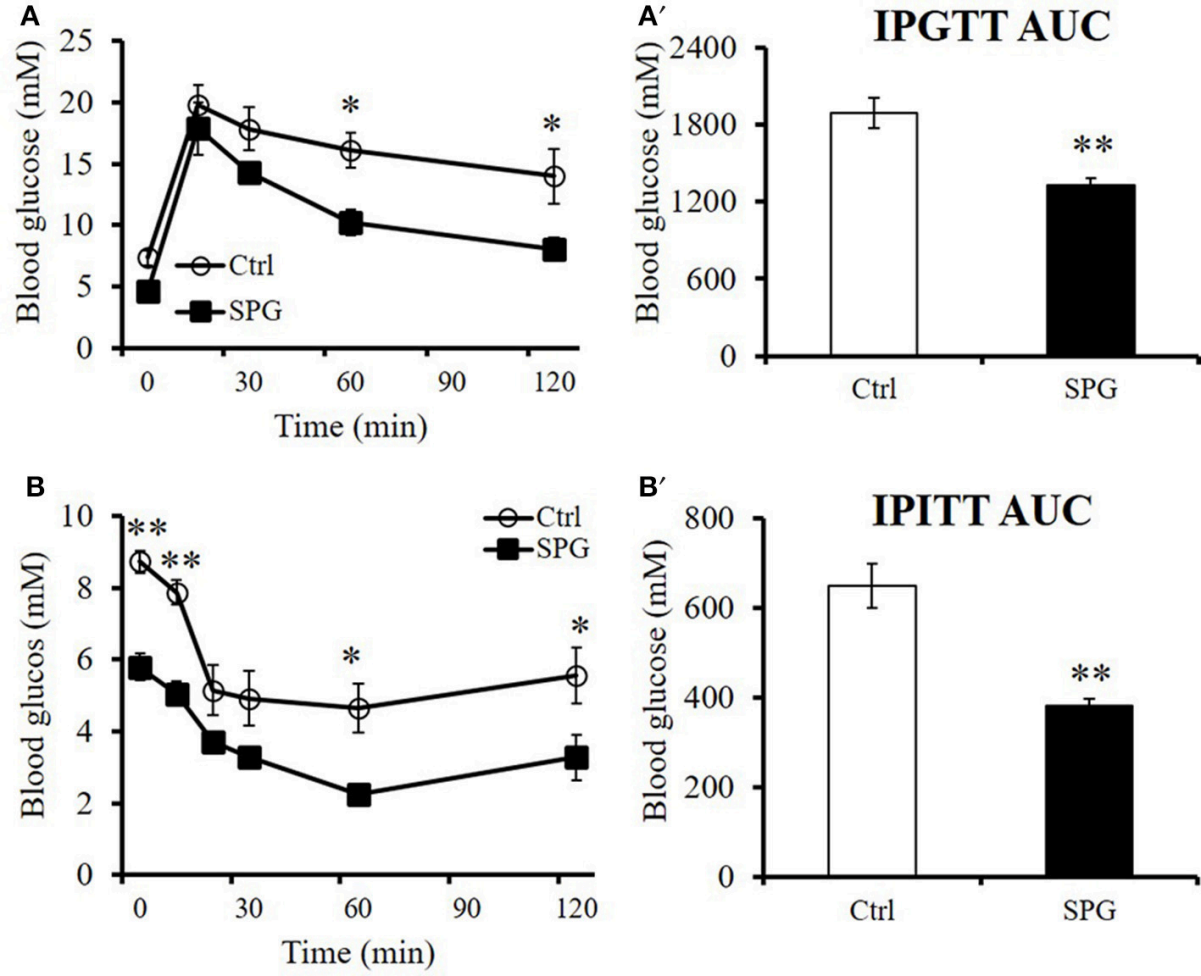
**Supaglutide improves glucose tolerance and insulin sensitivity in the DIO mice**. Glucose concentrations during **(A)** IPGTT or **(B)** an insulin tolerance test (ITT) in control and supaglutide-treated mice. The significant differences between groups in **(A)** and **(B)** are analyzed using two-way RM ANOVA method. Area under curve (AUC) for **(A')** IPGTT and **(B')** ITT were calculated. The significant differences between groups in **(A')** and **(B')** are analyzed using unpaired Student's *t*-test. All values are means ± SE (*n* = 5 for each group). ^*^p < 0.05; ^**^p < 0.01.

We have demonstrated that supaglutide has long-lasting effects on improving glucose tolerance of CD1 mice (Wang et al., 2010). In current study, we showed that supaglutide exerted dose-responsive effects in reducing glucose excursion in the euglycemic CD1 mice (Supplementary Figure 1). Furthermore, supaglutide at a dosage of 300 μg/kg was effective in improving glucose tolerance 216 h after a single drug-injection (Supplementary Figure 2). In order to determine the glucoregulatory effect of supaglutide on the diet-induced obese mice, we performed IPGTT and ITT in the obese mice, our studies showed that after the treatment for 4 weeks, the glucose tolerance and insulin sensitivity in the supaglutide-treated mice were significantly improved as compared to the control mice. Specifically, the treated obese mice showed lower level of fasting blood glucose and improved glucose excursion (Figure 9A) and improved insulin sensitivities (Figure 9B). When the glycemic response was expressed as AUC the changes were statistically different (Figure 9A', IPGTT: SPG vs. Ctrl = 1334.25 ± 19.86 vs. 1896.3 ± 53.58, *p* < 0.01; Figure 9B', ITT: SPG vs. Ctrl = 381.5 ± 7.63 vs. 648.13 ± 24.54, *p* < 0.01). Moreover, supaglutide was effective in reducing fasting blood glucose in the hyperglycemic db/db mice. Specifically, the drug-treated db/db mice exhibited a lower fasting blood glucose levels during 7 days' observations after a single dose injection (Supplementary Figure 3A). Interestingly, among doses tested, while supaglutide treatment showed dose-dependent effects on blood glucose lowering in the db/db mice, which was not observed in the context of its weight losing (i.e., lower dose tested showed better effects) (Supplementary Figure 3B). These results indicate that supaglutide exerted regulatory effects in energy homeostasis in the mice with established obesity and hyperglycemia.

## Discussion

The present study demonstrates that supaglutide administrated twice a week for 4 weeks in established diet-induced obese mice, resulted in an overall improvement in energy homeostasis, specifically, the significant reductions in body weight and amelioration of obesity-related metabolic disorders, including hyperglycemia, hyperlipidemia and hepatic steatosis.

Obesity is a major challenge for the prevention of metabolic diseases and complications associated with the disease. In recent years, there are increasing interests in the treatment of obesity by targeting GLP-1 signaling (DeFronzo et al., 2005; Astrup et al., 2009; Shah and Vella, 2014; Ladenheim, 2015; Isaacs et al., 2016). Notably, liraglutide, a GLP-1 analog, has become the first GLP-1 receptor targeting agent proved by FDA for management of obesity recently (Rajeev and Wilding, 2016). Clinical studies showed that this daily injecting agent is effective in reducing body weight of obese patients with or without T2D. The SCALE study involved 3731 patients without T2D showed that liraglutide at a once-daily dose of 3.0 mg, when used as an adjunct to a reduced-calorie diet and increased physical activity, increased weight loss in patients with a BMI of more than 30 or more than 27 if they had hypertension and dyslipidemia. The mean change in body weight with liraglutide was −8.0 ± 6.7% (−8.4 ± 7.3 kg) which was generally maintained over the course of the 56-week main study period, as long as the patients continued treatment (Pi-Sunyer et al., 2015). The LEAD1-6 series of trials showed that a once-daily dose of 1.2–1.8 mg of liraglutide yielded reductions of body weight by 2–3 kg in T2D (Rajeev and Wilding, 2016). Other GLP-1 analogs have also been found to be effective in treating obesity (Woodward and Anderson, 2014; Thompson and Trujillo, 2015). Specifically, the once a week dosing dulaglutide has demonstrated glycemic lowering effects and weight reduction similar to liraglutide and exenatide. Importantly, this once-weekly agent has demonstrated superior therapeutic effects when used as monotherapy compared with the twice-daily exenatide (Thompson and Trujillo, 2015). These finding highlighted that a strategy targeting GLP-1 signaling is clinically effective in treating obesity.

We have previously reported that supaglutide displayed distinguished PK profile and prolonged *in vivo* half-life (Wang et al., 2010). In the present study, we demonstrated that in established obese mouse model induced by HFD feeding; twice a week injection of supaglutide elicited a moderate but significant reduction of body weight by ~5%. The body weight sparing effects was consistently observed in db/db mice. It was noted that supaglutide at the tested doses showed dose-dependent effects in lowering blood glucose; however, this dose-response relationship was not found in its weight sparing effects in db/db mice, implying different mechanisms underlying the glucose-lowering and weight-reduction effects of supaglutide in db/db mice. Previous clinical studies demonstrated that a modest weight loss provides beneficial effects on cardiovascular risk factors in obese patients (Goldstein, 1992; Van Gaal et al., 1997). The outcome of present study that Supaglutide reduces body weight in obese mice implies a clinical relevance in the terms of metabolic disorders and cardiac beneficial effects.

For an ideal anti-obesity therapy, it is preferable that the weight loss stems predominantly from fat rather than lean tissues. We found that supaglutide induced decrease in body weight which was accompanied with a reduction in epididymal fat, which represents part of visceral fat. However, the weights of interscapular BAT and inguinal fat were not significantly changed. In general, visceral fat was considered as “bad fat” since it was associated with insulin resistance and increased cardiovascular risk (Hocking et al., 2013; Bouchi et al., 2016). However, subcutaneous fat and BAT were considered as “good fat” given their beneficial effects on the metabolism homeostasis (Hocking et al., 2013). It is presumably the reduction of epididymal fat may partly contribute to the beneficial effect of Supglutide in improving energy metabolism in the obese mice. In contrast, our result showed that the weights of lower leg muscles (including gastrocnemius and soleus) were not altered by supaglutide, indicating that the reduction in body weight of the obese mice was not due to losing lean mass.

We found that supaglutide treatment reduced the weight of liver. The reduced liver mass was associated with decreased hepatocyte fat deposition and TG content. Remarkably, supaglutide-reduced liver fat accumulation was also accompanied by decreased ALT and AST levels, suggesting enhanced hepatic protective capability from HFD induced liver injury. It has been shown that excessive lipid accumulation within hepatocytes is the main cause to hepatocyte injury characterized by increased ALT and AST concentrations (Magee et al., 2016). Previous studies showed that HFD induced hepatic lipid accumulation was associated with increased liver inflammation, elevated hepatic endoplasmic reticulum stress and activation relevant signaling pathways (Liu et al., 2014), in which the obese-related liver injury could be ameliorated by GLP-1 treatment or therapies using GLP-1 mimetic (Armstrong et al., 2016a,b; Bouchi et al., 2016; He et al., 2016; Valdecantos et al., 2016).

It is noted that supaglutide treatment elicited a general shift to smaller adipocyte sizes in all adipose depots detected, including BAT, inguinal WAT as well as epididymal WAT. To certain extent, the size of adipocyte generally reflects the metabolic function of adipocytes (Skurk et al., 2007). Although, it is not clear which absolute range of adipocytes sizes is metabolically harmful (Verhoef et al., 2013), it is generally accepted that a conversion of small adipocytes to large ones is closely related to common, health risks including hyperlipidemia, diabetes, hypertension and cardiovascular diseases. In a sharp contrast, an increase in the number of small adipocytes promotes lipid metabolism and insulin sensitivity (Spiegelman and Flier, 1996). Our observations that supaglutide decreased adipocyte size in the fat tissues tested were consistent with the improved energy metabolism in these obese mice.

Supaglutide therapy improved metabolic conditions in HFD-induced obese mice was associated with significant reduction in TC, TG, NEFA, and a trend of decreased LDL. Elevation of these dyslipidemia-related circulating parameters are considered to be predictive of heart disease and atherosclerosis in obese and diabetic populations (Mokdad et al., 2003). Recent studies revealed beneficial effects of GLP-1 and its metabolites in cardiac dysfunction, reviewed in Li et al. (2017). Given its long-lasting GLP-1 actions and potential clinical compliance, it is of great interests to investigate whether supaglutide exerts cardioprotective effects in both preclinical and clinical settings.

Reduction in body weight may be a consequence of lower food intake and/or enhanced energy expenditure. In current study, supaglutide therapy exerted anorectic effects in the obese mice. The energy expenditure of the mice was studied by indirect calorimetry using CLAMS. According to our data, VO2, VCO2, and EE expressed as per-whole-animal showed a trend, but not statistically different, of increase in supaglutide group. However, when normalized by BW, VO2, VCO2, and EE of the supaglutide-treated mice demonstrated significant increase in the dark cycle.

Whether the CLAMS values should be normalized remains a debate because sometimes simple division of CLAMS data by BW would lead to contradictory or contrary conclusion as compared to un-normalized data (Butler and Kozak, 2010). However, body size itself is a crucial but complex determinant of energy expenditure. Larger animals typically have higher absolute rates of energy expenditure owing to an increase in the total amount of metabolically active mass, therefore making it necessary to adjust EE data for the influence of body size variation *per se* (Kaiyala and Schwartz, 2011).

It should be noted, under certain circumstance the dynamic changes and adaptive response in EE during an intervention somehow adds to the difficulty in analyzing the metabolic effect of certain treatment. Specifically, even though certain treatment promotes weight reduction through enhancing energy expenditure at the beginning, the weight loss will probably lead to reduced EE in turn as an adaptive response to maintain the homeostasis of body weight, which is seen in most studies (Leibel et al., 1995; Keesey and Hirvonen, 1997; Wyatt et al., 1999; MacLean et al., 2004). In our current study, the mice were subjected to the CLAMS system for analysis of metabolic status at the end of the experiment, but the weight reduction effect was seen after the first drug injection and maintained throughout the experiment, rendering the possibility that we might have missed the best time window to quantify EE that significantly influences body weight gain. Nevertheless, our data that Supaglultide-treated mice demonstrated a concomitantly increased trend of EE both on per-whole-animal and BW-normalized basis suggested that supaglutide exerted a regulatory effect on the energy expenditure of the obese mice, which is in good agreement with previous studies in both preclinical and clinical obese subjects (Kanoski et al., 2011; Fukuda-Tsuru et al., 2014; Shah and Vella, 2014; Decara et al., 2016; Rondanelli et al., 2016; Xu et al., 2016). It is important to note, the tissue-specific modulation of lipid metabolism and WAT remodeling of GLP-1 represents a molecular mechanism in mediating GLP-1's anti-obesity actions (Decara et al., 2016; Xu et al., 2016).

WAT and BAT, the two major fat tissues are found different morphologically and functionally in mammals (Wu et al., 2015). Specifically, WAT is mainly responsible for energy storage and BAT is mainly for thermoregulation i.e., to dissipate chemical energy in the form of heat (Wu et al., 2015). While WAT is traditionally known as the biggest mammalian triacylglycerol storage depot, recent studies suggest that WAT can be rendered brown-like features in response to certain stimulus (Jeremic et al., 2016). Remodeling of WAT is characterized by up-regulation of the BAT-specific gene Ucp1 and therefore exerts an energy-disposal capacity (Wu et al., 2015; Jeremic et al., 2016). Central infusion of GLP-1 was reported to stimulate BAT thermogenesis (Lockie et al., 2012; Beiroa et al., 2014; Kooijman et al., 2015). However, the role of peripheral GLP-1R activation in regulating energy expenditure remains controversial (Beiroa et al., 2014). Our observations that chronic peripheral treatment with the novel GLP-1R agonist supaglutide upregulated the expression of Ucp1 in inguinal WAT, but not in BAT or epididymal WAT, suggesting increase in UCP1-containing adipocytes (Petrovic et al., 2010) or beige adipocytes (Wu et al., 2012) upon supaglutide treatment. It is conceivable that supaglutide-induced anti-obesity effect was at least in part contributed by the browning of inguinal WAT, rather than activated BAT thermogenesis. Mechanistic analysis in the context of white browning phenotype in supaglutide treated mice is clearly warranted for future study. However, our data is supported by others' studies which demonstrated that liraglutide treatment did not affect BAT thermogenesis, but significantly reduced body weight and adiposity in obese mice (Heppner et al., 2015), implying that there is additional physiological mechanism underlying the effect of GLP-1R agonists on weight reduction. Nevertheless, it is possible that supaglutide treatment altered the adipose tissue heterogeneity i.e., changes in depot-specific differences may give rise to different metabolic consequences under obese condition (Rosell et al., 2014; Kwok et al., 2016).

Activation of UCP1 in BAT is a key factor in maintaining core body temperature especially when it is exposed to cold environment (Cannon and Nedergaard, 2004). Our study showed that during 6 h of exposure to cold, body temperatures of the untreated mice dropped significantly, displaying impaired thermogenesis compared with the drug treated-mice, which kept higher rectal temperature throughout the experiment, suggesting that supaglutide was able to increase body adaptation to cold exposure by generating more heat.

In current study, we showed that supaglutide enhanced glucose excursion and insulin sensitivity in obese mice. To certain extent, the rate of glucose excursion (IPGTT) depends on body's responsiveness to insulin. We observed that supaglutide significantly decreased hepatic levels of NEFA in obese mice. Since elevated hepatic NEFA can cause liver insulin resistance (Hirabara et al., 2010), it is conceivable that improved lipid metabolism in the obese mice that contributed to improved insulin sensitivity and glucose metabolism in the obese mice.

In a summary, in the present study, we demonstrated that the long-lasting GLP-1 receptor agonist Supraglutide promoted body weight loss in already established obesity in mice. This was associated with improved obesity-related metabolic disorders including hyperglycemia, hyperlipidemia, and hepatic steatosis. Moreover, we found that the beneficial effect of supaglutide on metabolic condition was associated with suppressed food intake and browning remodeling of WAT. The prolonged half-life and metabolically beneficial effect of supaglutide provides an alternative new tool in studying GLP-1 biology and potentially a novel therapeutic target to treat obesity and its associated metabolic disorders.

## Author contributions

QW contributed to the conception and design of the study; YW contributed to performing the major body of the experiments and data analysis of the study; XB, JH and XZ contributed to supaglutide production and performing of CD-1 and db/db mice related experiments. QC and WL contributed to performing part of experiments. YW, XB, JH, XZ, WL, QC, DJ, ZW, RL, and QW contributed to analysis and interpretation of the data. YW and QW contributed to writing the article. All authors have revised the manuscript critically for important intellectual content and given final approval of the version to be published.

## Funding

This project was partially supported by grants from Yunnan Provincial Science and Technology Department, Yunnan Development and Reform Commission, Kunming Municipal Bureau of Human Resources and Social Security, and Shanghai Science and Technology Department.

### Conflict of interest statement

QW is an inventor of GLP-1 related patents and serves on the Scientific Advisory Board of Diamyd Medical, and a founder of Yinnuo Pharmaceutical Technology Co. Ltd. No other potential conflicts of interest relevant to this article were reported. The other authors declare that the research was conducted in the absence of any commercial or financial relationships that could be construed as a potential conflict of interest.

## References

[B1] Armstrong M. J. Gaunt P. Aithal G. P. Barton D. Hull D. Parker R.. (2016a). Liraglutide safety and efficacy in patients with non-alcoholic steatohepatitis (LEAN): a multicentre, double-blind, randomised, placebo-controlled phase 2 study. Lancet 387, 679–690. 10.1016/S0140-6736(15)00803-X 26608256

[B2] Armstrong M. J. Hull D. Guo K. Barton D. Hazlehurst J. M. Gathercole L. L.. (2016b). Glucagon-like peptide 1 decreases lipotoxicity in non-alcoholic steatohepatitis. J. Hepatol. 64, 399–408. 10.1016/j.jhep.2015.08.038 26394161 PMC4713865

[B3] Astrup A. Rössner S. Van Gaal L. Rissanen A. Niskanen L. Al Hakim M.. (2009). Effects of liraglutide in the treatment of obesity: a randomised, double-blind, placebo-controlled study. Lancet 374, 1606–1616. 10.1016/S0140-6736(09)61375-1 19853906

[B4] Beiroa D. Imbernon M. Gallego R. Senra A. Herranz D. Villarroya F.. (2014). GLP-1 agonism stimulates brown adipose tissue thermogenesis and browning through hypothalamic AMPK. Diabetes 63, 3346–3358. 10.2337/db14-0302 24917578

[B5] Bouchi R. Nakano Y. Fukuda T. Takeuchi T. Murakami M. Minami I.. (2016). Reduction of visceral fat by liraglutide is associated with ameliorations of hepatic steatosis, albuminuria, and micro-inflammation in type 2 diabetic patients with insulin treatment: a randomized control trial. Endocr. J. 64, 269–281. 10.1507/endocrj.EJ16-0449 27916783

[B6] Butler A. A. Kozak L. P. (2010). A recurring problem with the analysis of energy expenditure in genetic models expressing lean and obese phenotypes. Diabetes 59, 323–329. 10.2337/db09-1471 20103710 PMC2809965

[B7] Cannon B. Nedergaard J. (2004). Brown adipose tissue: function and physiological significance. Physiol. Rev. 84, 277–359. 10.1152/physrev.00015.2003 14715917

[B8] Decara J. Arrabal S. Beiroa D. Rivera P. Vargas A. Serrano A.. (2016). Antiobesity efficacy of GLP-1 receptor agonist liraglutide is associated with peripheral tissue-specific modulation of lipid metabolic regulators. Biofactors 42, 600–611. 10.1002/biof.1295 27213962

[B9] DeFronzo R. A. Ratner R. E. Han J. Kim D. D. Fineman M. S. Baron A. D. (2005). Effects of exenatide (exendin-4) on glycemic control and weight over 30 weeks in metformin-treated patients with type 2 diabetes. Diabetes Care 28, 1092–1100. 10.2337/diacare.28.5.1092 15855572

[B10] Drucker D. J. Dritselis A. Kirkpatrick P. (2010). Liraglutide. Nat. Rev. Drug Discov. 9, 267–268. 10.1038/nrd3148 20357801

[B11] Drucker D. J. Sherman S. I. Bergenstal R. M. Buse J. B. (2011). The safety of incretin-based therapies–review of the scientific evidence. J. Clin. Endocrinol. Metab. 96, 2027–2031. 10.1210/jc.2011-0599 21734003

[B12] Fukuda-Tsuru S. Kakimoto T. Utsumi H. Kiuchi S. Ishii S. (2014). The novel dipeptidyl peptidase-4 inhibitor teneligliptin prevents high-fat diet-induced obesity accompanied with increased energy expenditure in mice. Eur. J. Pharmacol. 723, 207–215. 10.1016/j.ejphar.2013.11.030 24309217

[B13] Goldstein D. J. (1992). Beneficial health effects of modest weight loss. Int. J. Obes. Relat. Metab. Disord. 16, 397–415. 1322866

[B14] He Q. Sha S. Sun L. Zhang J. Dong M. (2016). GLP-1 analogue improves hepatic lipid accumulation by inducing autophagy via AMPK/mTOR pathway. Biochem. Biophys. Res. Commun. 476, 196–203. 10.1016/j.bbrc.2016.05.086 27208776

[B15] Heppner K. M. Marks S. Holland J. Ottaway N. Smiley D. Dimarchi R.. (2015). Contribution of brown adipose tissue activity to the control of energy balance by GLP-1 receptor signalling in mice. Diabetologia 58, 2124–2132. 10.1007/s00125-015-3651-3 26049402 PMC4529364

[B16] Hirabara S. M. Curi R. Maechler P. (2010). Saturated fatty acid-induced insulin resistance is associated with mitochondrial dysfunction in skeletal muscle cells. J. Cell. Physiol. 222, 187–194. 10.1002/jcp.21936 19780047

[B17] Hocking S. Samocha-Bonet D. Milner K. L. Greenfield J. R. Chisholm D. J. (2013). Adiposity and insulin resistance in humans: the role of the different tissue and cellular lipid depots. Endocr. Rev. 34, 463–500. 10.1210/er.2012-1041 23550081

[B18] Hupe-Sodmann K. McGregor G. P. Bridenbaugh R. Göke R. Göke B. Thole H.. (1995). Characterisation of the processing by human neutral endopeptidase 24.11 of GLP-1(7-36) amide and comparison of the substrate specificity of the enzyme for other glucagon-like peptides. Regul. Pept. 58, 149–156. 8577927 10.1016/0167-0115(95)00063-h

[B19] Isaacs D. Prasad-Reddy L. Srivastava S. B. (2016). Role of glucagon-like peptide 1 receptor agonists in management of obesity. Am. J. Health Syst. Pharm. 73, 1493–1507. 10.2146/ajhp150990 27521241

[B20] Jeremic N. Chatuverdi P. Tyagi S. C. (2016). Browning of white fat: novel insight into factors, mechanisms and therapeutics. J. Cell. Physiol. 232, 61–68. 10.1002/jcp.25450 27279601 PMC6567990

[B21] Kaiyala K. J. Schwartz M. W. (2011). Toward a more complete (and less controversial) understanding of energy expenditure and its role in obesity pathogenesis. Diabetes 60, 17–23. 10.2337/db10-0909 21193735 PMC3012169

[B22] Kanoski S. E. Fortin S. M. Arnold M. Grill H. J. Hayes M. R. (2011). Peripheral and central GLP-1 receptor populations mediate the anorectic effects of peripherally administered GLP-1 receptor agonists, liraglutide and exendin-4. Endocrinology 152, 3103–3112. 10.1210/en.2011-0174 21693680 PMC3138234

[B23] Keesey R. E. Hirvonen M. D. (1997). Body weight set-points: determination and adjustment. J. Nutr. 127, 1875S–1883S. 9278574 10.1093/jn/127.9.1875S

[B24] Kieffer T. J. McIntosh C. H. Pederson R. A. (1995). Degradation of glucose-dependent insulinotropic polypeptide and truncated glucagon-like peptide 1 *in vitro* and *in vivo* by dipeptidyl peptidase IV. Endocrinology 136, 3585–3596. 10.1210/endo.136.8.7628397 7628397

[B25] Kooijman S. Wang Y. Parlevliet E. T. Boon M. R. Edelschaap D. Snaterse G.. (2015). Central GLP-1 receptor signalling accelerates plasma clearance of triacylglycerol and glucose by activating brown adipose tissue in mice. Diabetologia 58, 2637–2646. 10.1007/s00125-015-3727-0 26254578 PMC4589565

[B26] Kumar M. Hunag Y. Glinka Y. Prud'homme G. J. Wang Q. (2007). Gene therapy of diabetes using a novel GLP-1/IgG1-Fc fusion construct normalizes glucose levels in db/db mice. Gene Ther. 14, 162–172. 10.1038/sj.gt.3302836 16943856

[B27] Kwok K. H. Lam K. S. Xu A. (2016). Heterogeneity of white adipose tissue: molecular basis and clinical implications. Exp. Mol. Med. 48:e215. 10.1038/emm.2016.5 26964831 PMC4892883

[B28] Ladenheim E. E. (2015). Liraglutide and obesity: a review of the data so far. Drug Des. Devel. Ther. 9, 1867–1875. 10.2147/DDDT.S58459 25848222 PMC4386791

[B29] Lee C. Y. (2016). Glucagon-like peptide-1 formulation–the present and future development in diabetes treatment. Basic Clin. Pharmacol. Toxicol. 118, 173–180. 10.1111/bcpt.12524 26551045

[B30] Leibel R. L. Rosenbaum M. Hirsch J. (1995). Changes in energy expenditure resulting from altered body weight. N. Engl. J. Med. 332, 621–628. 10.1056/NEJM199503093321001 7632212

[B31] Li J. Zheng J. Wang S. Lau H. K. Fathi A. Wang Q. (2017). Cardiovascular benefits of native GLP-1 and its metabolites: an indicator for GLP-1-therapy strategies. Front. Physiol. 8:15. 10.3389/fphys.2017.00015 28194113 PMC5276855

[B32] Lindamood C. A. Taylor J. R. (2015). Emerging new therapies for the treatment of type 2 diabetes mellitus: glucagon-like peptide-1 receptor agonists. Clin. Ther. 37, 483–493. 10.1016/j.clinthera.2015.01.003 25659912

[B33] Liu J. Zhuang Z. J. Bian D. X. Ma X. J. Xun Y. H. Yang W. J.. (2014). Toll-like receptor-4 signalling in the progression of non-alcoholic fatty liver disease induced by high-fat and high-fructose diet in mice. Clin. Exp. Pharmacol. Physiol. 41, 482–488. 10.1111/1440-1681.12241 24739055

[B34] Lockie S. H. Heppner K. M. Chaudhary N. Chabenne J. R. Morgan D. A. Veyrat-Durebex C.. (2012). Direct control of brown adipose tissue thermogenesis by central nervous system glucagon-like peptide-1 receptor signaling. Diabetes 61, 2753–2762. 10.2337/db11-1556 22933116 PMC3478556

[B35] MacLean P. S. Higgins J. A. Johnson G. C. Fleming-Elder B. K. Donahoo W. T. Melanson E. L.. (2004). Enhanced metabolic efficiency contributes to weight regain after weight loss in obesity-prone rats. Am. J. Physiol. Regul. Integr. Comp. Physiol. 287, R1306–R1315. 10.1152/ajpregu.00463.2004 15331386

[B36] Magee N. Zou A. Zhang Y. (2016). Pathogenesis of nonalcoholic steatohepatitis: interactions between liver parenchymal and nonparenchymal cells. Biomed Res. Int. 2016:5170402. 10.1155/2016/5170402 27822476 PMC5086374

[B37] Meier J. J. Nauck M. A. (2005). Glucagon-like peptide 1(GLP-1) in biology and pathology. Diabetes Metab. Res. Rev. 21, 91–117. 10.1002/dmrr.538 15759282

[B38] Mokdad A. H. Ford E. S. Bowman B. A. Dietz W. H. Vinicor F. Bales V. S.. (2003). Prevalence of obesity, diabetes, and obesity-related health risk factors, 2001. JAMA 289, 76–79. 10.1001/jama.289.1.76 12503980

[B39] Petrovic N. Walden T. B. Shabalina I. G. Timmons J. A. Cannon B. Nedergaard J. (2010). Chronic peroxisome proliferator-activated receptor gamma (PPARgamma) activation of epididymally derived white adipocyte cultures reveals a population of thermogenically competent, UCP1-containing adipocytes molecularly distinct from classic brown adipocytes. J. Biol. Chem. 285, 7153–7164. 10.1074/jbc.M109.053942 20028987 PMC2844165

[B40] Pi-Sunyer X. Astrup A. Fujioka K. Greenway F. Halpern A. Krempf M.. (2015). A randomized, controlled trial of 3.0 mg of liraglutide in weight management. N. Engl. J. Med. 373, 11–22. 10.1056/NEJMoa1411892 26132939

[B41] Rajeev S. P. Wilding J. (2016). GLP-1 as a target for therapeutic intervention. Curr. Opin. Pharmacol. 31, 44–49. 10.1016/j.coph.2016.08.005 27591964

[B42] Rondanelli M. Perna S. Astrone P. Grugnetti A. Solerte S. B. Guido D. (2016). Twenty-four-week effects of liraglutide on body composition, adherence to appetite, and lipid profile in overweight and obese patients with type 2 diabetes mellitus. Patient Prefer. Adherence 10, 407–413. 10.2147/PPA.S97383 27069358 PMC4818054

[B43] Rosell M. Kaforou M. Frontini A. Okolo A. Chan Y. W. Nikolopoulou E.. (2014). Brown and white adipose tissues: intrinsic differences in gene expression and response to cold exposure in mice. Am. J. Physiol. Endocrinol. Metab. 306, E945–E964. 10.1152/ajpendo.00473.2013 24549398 PMC3989735

[B44] Shah M. Vella A. (2014). Effects of GLP-1 on appetite and weight. Rev. Endocr. Metab. Disord. 15, 181–187. 10.1007/s11154-014-9289-5 24811133 PMC4119845

[B45] Skurk T. Alberti-Huber C. Herder C. Hauner H. (2007). Relationship between adipocyte size and adipokine expression and secretion. J. Clin. Endocrinol. Metab. 92, 1023–1033. 10.1210/jc.2006-1055 17164304

[B46] Soltani N. Kumar M. Glinka Y. Prud'homme G. J. Wang Q. (2007). *In vivo* expression of GLP-1/IgG-Fc fusion protein enhances beta-cell mass and protects against streptozotocin-induced diabetes. Gene Ther. 14, 981–988. 10.1038/sj.gt.3302944 17410180

[B47] Spiegelman B. M. Flier J. S. (1996). Adipogenesis and obesity: rounding out the big picture. Cell 87, 377–389. 8898192 10.1016/s0092-8674(00)81359-8

[B48] Stanford K. I. Middelbeek R. J. Townsend K. L. An D. Nygaard E. B. Hitchcox K. M.. (2013). Brown adipose tissue regulates glucose homeostasis and insulin sensitivity. J. Clin. Invest. 123, 215–223. 10.1172/JCI62308 23221344 PMC3533266

[B49] Thompson A. M. Trujillo J. M. (2015). Dulaglutide: the newest GLP-1 receptor agonist for the management of type 2 diabetes. Ann. Pharmacother. 49, 351–359. 10.1177/1060028014564180 25565404

[B50] Tseng Y. H. Kokkotou E. Schulz T. J. Huang T. L. Winnay J. N. Taniguchi C. M.. (2008). New role of bone morphogenetic protein 7 in brown adipogenesis and energy expenditure. Nature 454, 1000–1004. 10.1038/nature07221 18719589 PMC2745972

[B51] Valdecantos M. P. Pardo V. Ruiz L. Castro-Sánchez L. Lanzón B. Fernández-Millán E.. (2016). A novel glucagon-like peptide 1/glucagon receptor dual agonist improves steatohepatitis and liver regeneration in mice. Hepatology 65, 950–968. 10.1002/hep.28962 27880981

[B52] Van Gaal L. F. Wauters M. A. De Leeuw I. H. (1997). The beneficial effects of modest weight loss on cardiovascular risk factors. Int. J. Obes. Relat. Metab. Disord. 21(Suppl. 1), S5–S9. 9130034

[B53] Verhoef S. P. van Dijk P. Westerterp K. R. (2013). Relative shrinkage of adipocytes by paraffin in proportion to plastic embedding in human adipose tissue before and after weight loss. Obes. Res. Clin. Pract. 7, e8–e13. 10.1016/j.orcp.2012.03.001 24331678

[B54] Wan Xue R. Wang Zhang Q. Huang S. Wu W. (2015). The effect of neuropeptide Y on brown-like adipocyte's differentiation and activation. Peptides 63, 126–133. 10.1016/j.peptides.2014.10.018 25451330

[B55] Wang Q. Chen K. Liu R. Zhao F. Gupta S. Zhang N.. (2010). Novel GLP-1 fusion chimera as potent long acting GLP-1 receptor agonist. PLoS ONE 5:e12734. 10.1371/journal.pone.0012734 20856794 PMC2939854

[B56] Woodward H. N. Anderson S. L. (2014). Once-weekly albiglutide in the management of type 2 diabetes: patient considerations. Patient Prefer. Adherence 8, 789–803. 10.2147/PPA.S53075 24926194 PMC4049886

[B57] Wu J. Boström P. Sparks L. M. Ye L. Choi J. H. Giang A. H.. (2012). Beige adipocytes are a distinct type of thermogenic fat cell in mouse and human. Cell 150, 366–376. 10.1016/j.cell.2012.05.016 22796012 PMC3402601

[B58] Wu J. Jun H. McDermott J. R. (2015). Formation and activation of thermogenic fat. Trends Genet. 31, 232–238. 10.1016/j.tig.2015.03.003 25851693 PMC4416987

[B59] Wyatt H. R. Grunwald G. K. Seagle H. M. Klem M. L. McGuire M. T. Wing R. R.. (1999). Resting energy expenditure in reduced-obese subjects in the National weight control registry. Am. J. Clin. Nutr. 69, 1189–1193. 10357738 10.1093/ajcn/69.6.1189

[B60] Xu F. Lin B. Zheng X. Chen Z. Cao H. Xu H.. (2016). GLP-1 receptor agonist promotes brown remodelling in mouse white adipose tissue through SIRT1. Diabetologia 59, 1059–1069. 10.1007/s00125-016-3896-5 26924394

