# Supplementary material for: Novel GLP-1 Analog Supaglutide Reduces HFD-Induced Obesity Associated with Increased Ucp-1 in White Adipose Tissue in Mice
Source: Front Physiol. 2017 May 15;8:294. doi: 10.3389/fphys.2017.00294 (PMC5430033; doi:10.3389/fphys.2017.00294)

Supplementary Fig. 1 SPG exerts dose-dependent effect on improving glucose tolerance in CD-1 mice

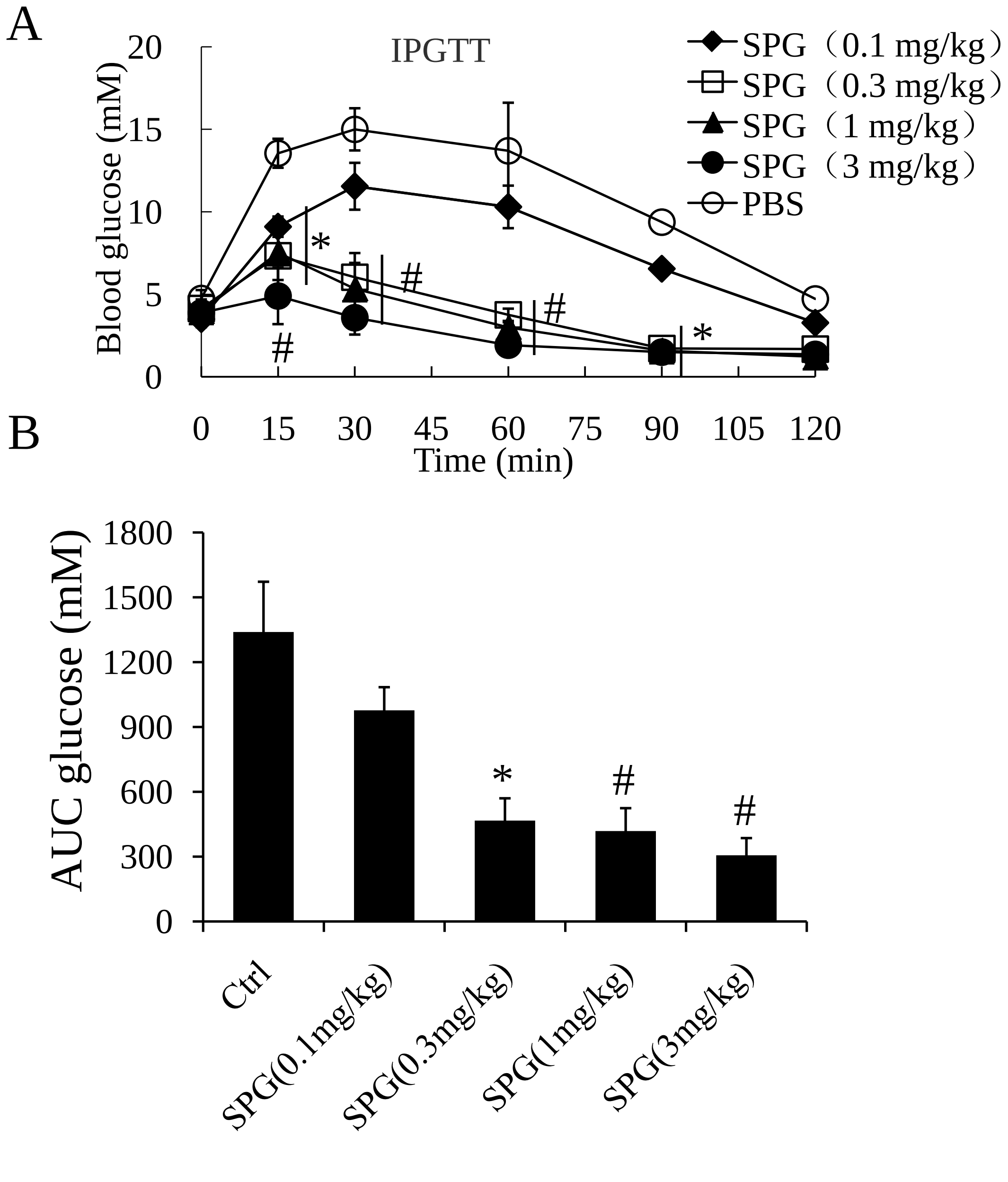

Supplementary Fig. 2 SPG has long-lasting effects on regulating glucose tolerance in CD-1 mice

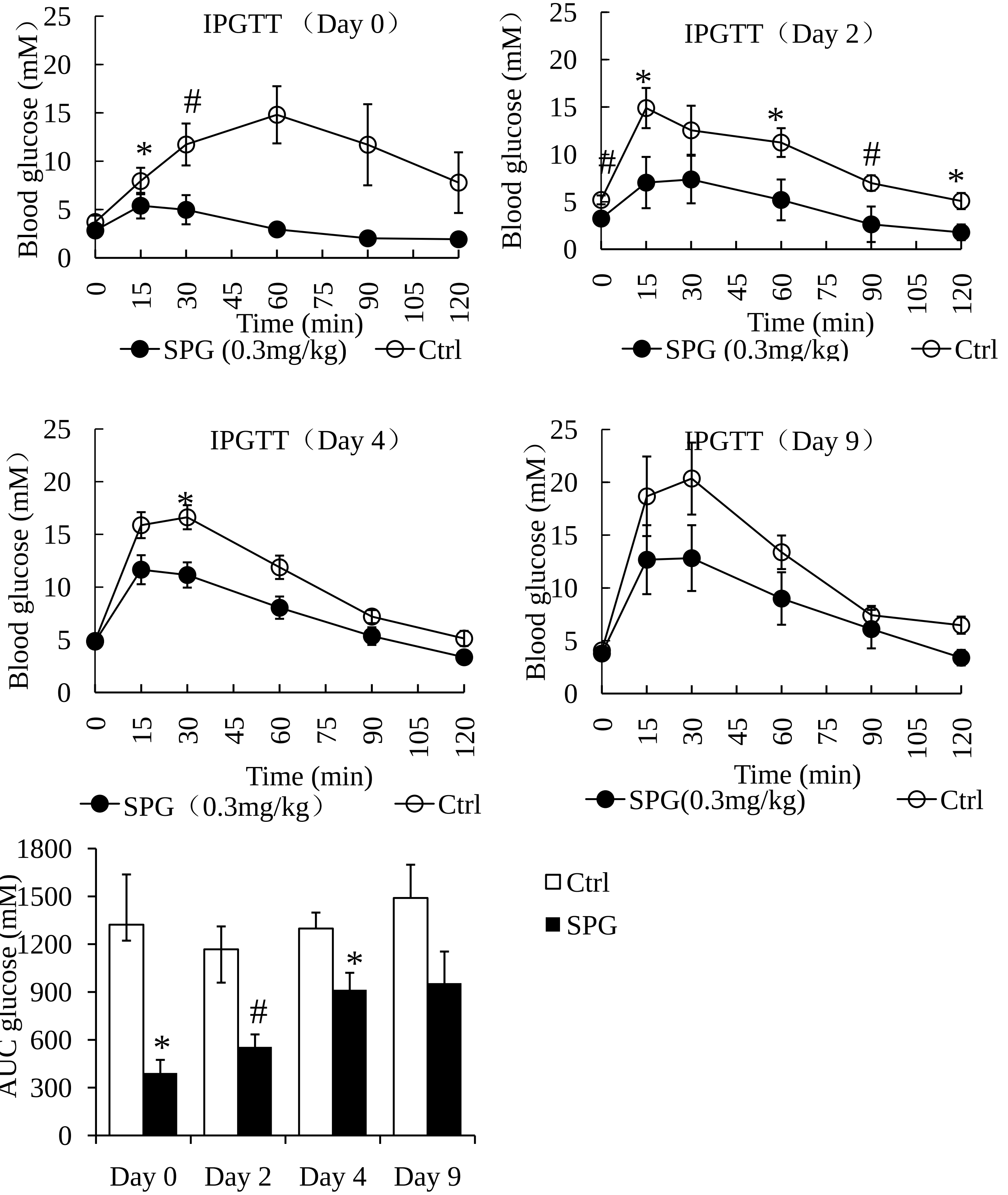

Supplementary Fig. 3 SPG reduces fasting blood glucose and body weight gain in db/db mice

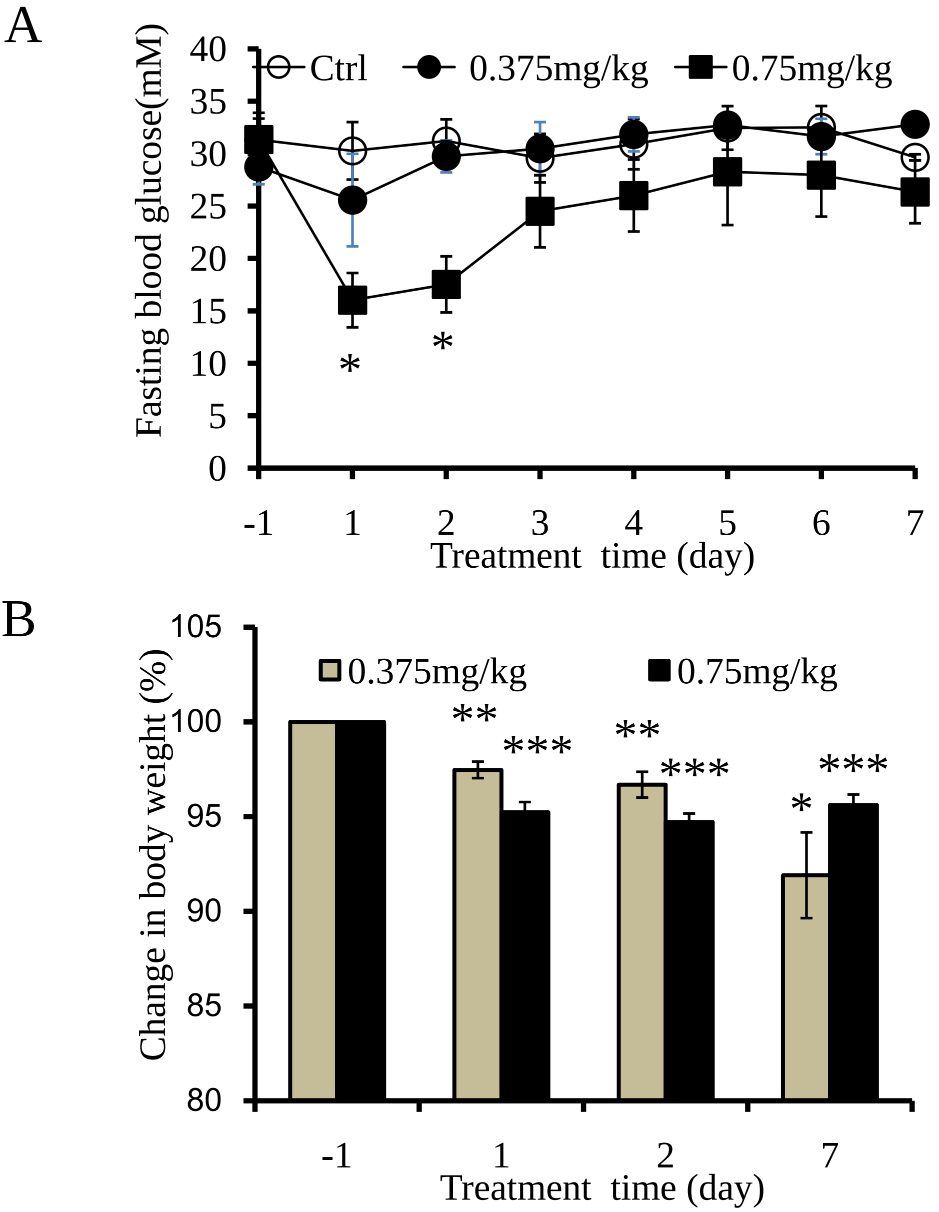

Supplement: Supplementary file 1 [file Presentation1.pdf]
