# Supplementary material for: Novel GLP-1 Analog Supaglutide Reduces HFD-Induced Obesity Associated with Increased Ucp-1 in White Adipose Tissue in Mice
Source: Front Physiol. 2017 May 15;8:294. doi: 10.3389/fphys.2017.00294 (PMC5430033; doi:10.3389/fphys.2017.00294)
Supplement: Supplementary file 2 [file Presentation2.PDF]

Supplementary data

Fig.1 Dose-dependent effects of Supaglutide on glucose tolerance of CD-1 mice.

CD-1 mice were fed on chow diet for 8 weeks. (A)Glucose concentrations at 0 min were measured before the mice were treated with varied doses of Supaglutide (0.1mg/kg, 0.3 mg/kg, 1mg/kg and 3mg/kg) and PBS as control. Thirty minutes after Supaglutide injection, the mice were injected with 3g /kg of glucose and subjected to IPGTT as described in Methods. (B) Areas under the curves for glucose were calculated. Results are means ± SE (n = 5) and *P < 0.05 vs. Ctrl, #P < 0.01 vs. Ctrl.

Fig. 2 SPG has long-lasting effects on regulating glucose tolerance in CD-1 mice

CD-1 mice at 7 weeks of age were injected with 0.3mg/kg Supaglutide before subjected to IPGTT at (A) Day 0 (30min after drug injection)、(B) Day 2、(C) Day 4 and (D) Day 9. (E)Area under the curve for glucose was calculated. Results are means ± SE (n = 4) and *P < 0.05 vs. Ctrl, # P < 0.01 vs. Ctrl.

Fig. 3 SPG reduces fasting blood glucose and body weight gain in db/db mice.

The db/db mice were fed on standard chow for 7 weeks followed by subcutaneous injection of Supaglutide (0.375 mg/kg and 0.75 mg/kg) or PBS as control. The day of drug injection was defined as day0. The basal fasting blood glucose (A) and body weight (B) were measured 1 day before injection (day-1) and recorded for 7 continuous days (day1 to day7) after the drug injection at day0. Basal Body weights of the mice were expressed as 100%， the changes in body weights after drug injection were compared to their basal body weights. Results are means ± SE (n = 3) and *P < 0.05 , **P < 0.01.
